# PumpSpectra: An MCSA-Based Platform for Fault Detection in Centrifugal Pump Systems

**DOI:** 10.3390/s25226916

**Published:** 2025-11-12

**Authors:** Hamza Adaika, Zoheir Tir, Mohamed Sahraoui, Khaled Laadjal

**Affiliations:** 1 LEVRES Laboratory, University of El Oued, El Oued 39000, Algeria; hamza-adaika@univ-eloued.dz (H.A.); tir-zoheir@univ-eloued.dz (Z.T.); 2 LGEB Laboratory, University of Biskra, Biskra 07000, Algeria; m.sahraoui@univ-biskra.dz; 3 IREC—Catalonia Institute for Energy Research, Jardins de les Dones de Negre 1, planta 2, 08930 Sant Adrià del Besòs, Barcelona, Spain

**Keywords:** motor current signature analysis, centrifugal pump, fault detection, predictive maintenance, spectral analysis, induction motor, misalignment defects, industrial monitoring

## Abstract

**Highlights:**

**What are the main findings?**

- PumpSpectra achieved 91.2% accuracy and rapid diagnostics for centrifugal pump faults in industry.
- It introduces an explainable, rule-based platform with severity grading for major pump faults.

**What is the implication of the main finding?**

- Enables cost-saving, sensor-minimal predictive maintenance for pump assets.
- Supports transparent, trusted decisions for industrial maintenance teams.

**Abstract:**

Reliable detection of faults in centrifugal pump systems is challenging in industrial environments due to harsh operating conditions, limited sensor access, and the need for fast, explainable decisions. We developed PumpSpectra, an industrial Motor Current Signature Analysis (MCSA) platform that processes uploaded stator-current CSV files using FFT/STFT with transparent, rule-based models designed to identify mechanical faults including misalignment, bearing defects, and impeller anomalies; field validation demonstrated misalignment detection. In a case study at the El Oued desalination plant (Algeria; n=40 operating points), PumpSpectra achieved 91.2% diagnostic accuracy with a 95% reduction in analysis time compared to manual MCSA post-processing, and a false-positive rate of 3.8% at 0.1 Hz resolution. These results suggest that current-only, explainable analytics can support predictive maintenance programs by accelerating fault triage, improving traceability of decisions, and reducing avoided maintenance costs in pump-driven industrial assets.

## 1. Introduction

Condition monitoring and predictive maintenance are critical in industrial plants where three-phase induction motors drive centrifugal pumps. These pumps are widely deployed in water supply, desalination, and wastewater treatment facilities; failures caused by bearing wear, impeller damage, hydraulic blockage, or shaft misalignment can trigger costly downtime and safety risks [1,2]. Traditional diagnostics typically rely on vibration sensors and manual inspections, which, while effective, are intrusive and difficult to scale plant-wide [1,2]. To address these constraints, electrical methods have gained traction.

In this work, we use Motor Current Signature Analysis (MCSA), the extraction and interpretation of characteristic frequency components from stator current signals as a non-invasive and cost-effective diagnostic approach. Throughout this paper, we use the term MCSA consistently to denote current-only analysis of induction motor–pump trains using Fast Fourier Transform (FFT) and Short-Time Fourier Transform (STFT); characteristic sidebands around the supply fundamental and its time and space harmonics reflect both electrical faults and pump-induced mechanical/hydraulic phenomena [3,4]. Unlike vibration-based methods, MCSA requires only clamp-on current measurements, avoiding additional instrumentation or disassembly and enabling retrofit deployments [1,2]. Recent studies extend MCSA to pump-specific anomalies beyond classic motor defects (broken rotor bars, inter-turn shorts, eccentricity, bearing faults). For off-design operation and cavitation, interpretable spectral indicators in the stator-current spectrum have been derived and experimentally validated [3,4]. In wastewater submersibles, impeller clogging alters specific current-spectrum lines, enabling threshold-based detection in both stationary and transient regimes [5]. Station-level cavitation detection via MCSA has been reported in pumped-storage systems [6], and rotational-frequency estimation from current has been used to flag broken impellers [7]. Multi-sensor lab studies combining vibration with current suggest improved reliability for grading cavitation severity and detecting off-design states [8]. Field-facing efforts now include multi-site, current-only data collection with IoT pipelines and Machine Learning (ML) classifiers over DQ/Concordia patterns [9].

While these studies advance the science of MCSA, most implementations are laboratory-controlled or research prototypes. While previous studies focused on laboratory-controlled MCSA implementations, little attention has been given to user-friendly, transparent diagnostic platforms suitable for industrial deployment in harsh environments like desalination plants. Consequently, slip-aware electrical order tracking for Variable-Frequency Drives (VFDs), transparent rule-based severity grading tied to explicit sidebands, and standardized benchmarking with operational Key Performance Indicators (KPIs) remain rare in the literature [2,3,4,5,8]. These gaps motivate a user-facing, explainable, FFT/STFT-primary platform with traceable rules and exportable results.

To address these challenges, this paper presents PumpSpectra, a modular diagnostic platform for centrifugal pump systems driven by induction motors. PumpSpectra integrates offline signal uploading, spectral analysis (FFT/STFT), and automated rule-based detection grounded in established fault-frequency models. The platform aims to

- Facilitate modular and accessible fault analysis for pump-driven induction motors.
- Provide user-friendly visualization of spectral data for technicians and engineers.
- Automate detection and severity assessment using traceable, sideband-based rules.
- Support slip-aware electrical order tracking for VFD operation.
- Validate fault models through real-world deployment at the El Oued desalination plant.

By bridging physically grounded MCSA models with practical field-level diagnostics, PumpSpectra contributes to explainable and scalable predictive maintenance in pump-driven industrial systems.

## 2. Related Work and Theoretical Background

The diagnosis of faults in mechanical systems, particularly for centrifugal pumps, has been a pivotal area of research due to the critical nature of these components in industrial applications. Fault diagnosis approaches can generally be classified into two categories—vibration-based and current-based methods—each with unique advantages and limitations.

### 2.1. Review of Fault Diagnosis Approaches: Vibration-Based vs. Current-Based

Vibration-based methods have been widely adopted for fault diagnosis due to their ability to provide detailed insights into mechanical conditions. Techniques such as FFT, STFT, and wavelet transforms are commonly employed to analyze vibration signals and detect anomalies in mechanical systems. Recent studies have illustrated the effectiveness of Convolutional Neural Networks (CNNs) in enhancing the accuracy of fault detection in vibration signals, such as those demonstrated by Chen et al. in their work on railway point machines [10]. On the other hand, current-based diagnosis, particularly through MCSA, leverages the electrical signals of motors to infer mechanical faults. This method provides a non-intrusive means of monitoring machine health. Studies, such as the one by Wang et al., illustrate how current measurements can effectively identify rolling bearing faults through advanced signal processing techniques [11]. While traditional methods like vibration analysis may be more common, current-based methods present a distinct advantage in applications where accessibility is limited or where vibration sensors cannot be reliably installed [12].

### 2.2. Signal Processing Methods

Various signal processing techniques serve as the backbone for both vibration and current-based fault diagnosis. FFT is favored for its simplicity in transforming time domain signals into frequency domain representations, allowing for the identification of fault frequencies such as Bearing Pulse Frequency (BPF) and Ball Pass Frequency Inner (BPFI), which are indicative of specific types of mechanical failures [13]. Wavelets and Empirical Mode Decomposition have also gained attention due to their effectiveness in analyzing non-stationary signals, which are commonly encountered in real-world applications [14,15]. ML has emerged as a transformative approach for fault diagnosis, increasing the efficacy of traditional signal processing methods by enabling automated feature extraction and classification. For example, Chen et al. utilized CNNs to monitor bearing vibration signals, achieving significant improvements in diagnostic accuracy [10]. The integration of ML not only enhances the diagnostic capabilities but also provides a systemic approach to processing large datasets typical in industrial environments [13,16]. While learning-based methods offer strong pattern-recognition capabilities, their deployment in field settings is often constrained by dataset size, labeling costs, and reduced interpretability; the rule-based design in this study intentionally prioritizes explainability and rapid technician-facing feedback while remaining compatible with future ML augmentation.

#### Recent MCSA Developments

Recent research demonstrates significant advances in MCSA applications. Sunal et al. report high-accuracy fault detection using CNN-based analysis of current-derived features in pumps deployed across multiple sites, validating MCSA’s effectiveness under real-world conditions [9]. Han et al. develop model-based voltage and current analysis for torque oscillation detection that improves condition monitoring of centrifugal pumps [17]. Sun et al. show that MCSA can detect cavitation in watermaker systems, extending current-based diagnostics to hydraulic phenomena [6]. In parallel, Ventura et al. propose real-time MCSA pipelines for edge monitoring, which informed our choice of a modular, upload-based deployment architecture and the roadmap for a low-latency inference pipeline [18].

### 2.3. Limitations of Existing Tools

Despite the advancements in both vibration and current-based methodologies, several limitations persist. Vibration analysis can be sensitive to environmental noise and requires calibration for accurate fault identification [19]. Additionally, the installation and maintenance of vibration sensors can be cumbersome in complex machinery setups. Current-based methods, while offering a non-invasive alternative, may fail to capture faults that do not produce a significant electrical anomaly, such as some types of misalignment or cavitation [20].

Furthermore, existing diagnostic tools often lack comprehensive fault detection capabilities, meaning multiple methods may be needed to attain a holistic understanding of a pump’s condition. The need for a unified platform that combines both fault detection and predictive maintenance is critical as industries move towards more resilient operational frameworks.

Commercial MCSA platforms also suffer from several practical limitations: (1) black-box algorithms with little or no diagnostic transparency; (2) proprietary data formats that restrict portability and integration into an enterprise computerized maintenance management system (CMMS); (3) high licensing and maintenance costs (often USD 10,000–50,000 annually); and (4) limited customization for domain-specific applications (e.g., desalination pumps with VFDs). These limitations motivated PumpSpectra’s open and transparent approach with explainable rules and exportable results.

### 2.4. MCSA Fault Frequency Models

#### 2.4.1. Bearing Fault Frequencies

For rolling-element bearings, characteristic frequencies are given by

(1) $$\begin{array}{cc} f_{\mathrm{BPFO}} & =\frac{N_{b}}{2}\;f_{r}\left(1-\frac{d}{D}\cos\phi \right), \end{array}$$

(2) $$\begin{array}{cc} f_{\mathrm{BPFI}} & =\frac{N_{b}}{2}\;f_{r}\left(1+\frac{d}{D}\cos\phi \right), \end{array}$$

(3) $$\begin{array}{cc} f_{\mathrm{BSF}} & =\frac{D}{2d}\;f_{r}\left(1-{\left(\frac{d}{D}\cos\phi \right)}^{2}\right), \end{array}$$

where $N_{b}$ is the number of rolling elements (balls), $f_{r}$ the mechanical rotation frequency, *d* the ball diameter, *D* the bearing pitch diameter, and ϕ the contact angle. These relations underpin the overlay of theoretical lines in PumpSpectra’s spectra for transparent interpretation.

The efficacy of fault diagnosis relies on established theoretical frequency models that correlate specific mechanical faults with distinct frequency signatures. For instance, rotor bar failures can produce unique fault frequencies that can be monitored through MCSA, while faults in bearings can be represented through empirical models linking them to Ball Spin Frequency (BSF), BPFI, and Ball Pass Frequency Outer (BPFO) [21]. These models form the foundation upon which diagnostic techniques are built and are essential for the accurate identification and categorization of faults in mechanical systems.

#### 2.4.2. Reference Fault Models

For completeness, the dominant spectral relations exploited by PumpSpectra are summarized below. Turbine blade rotation introduces sidebands at

(4) $$f_{\mathrm{turb}}=f_{s}\pm k\;N_{L}\;f_{r},$$

with k∈Z and $N_{L}$ the number of blades.

Bearing race defects yield

(5) $$f_{\mathrm{bague},\mathrm{int}}=f_{s}\pm k\times 0.6\times n_{b}\times f_{r},$$

(6) $$f_{\mathrm{bague},\mathrm{ext}}=f_{s}\pm k\times 0.4\times n_{b}\times f_{r},$$

where $n_{b}$ is the ball count. Angular or parallel misalignment produces

(7) $$f_{\mathrm{désalig}}=f_{s}\pm k\;f_{r}.$$

Together, these reference relations frame the diagnostic context for the performance and usability assessments discussed in the next section.

In conclusion, while both vibration-based and current-based fault diagnosis approaches have their respective merits and demerits, an integrated approach that combines the strengths of each methodology can enhance diagnostic robustness (Table 1). The PumpSpectra platform aims to provide an interpretable, rule-based approach with asset-agnostic thresholds, designed for rapid field deployment and clear visualization. The main research gap addressed is the lack of tools that combine traceable, explainable rules with practical, site-tested spectral automation—bridging the divide between black-box ML models and overly manual spectral analysis. This is especially important for industrial sites like the El Oued desalination plant, where labeled data are scarce, and interpretability and deployment speed are prioritized.

## 3. System Architecture and Implementation

PumpSpectra was designed as a modular diagnostic platform that integrates data acquisition, signal processing, fault detection, and reporting into a transparent and user-friendly framework. The architecture follows a sequential workflow, from current signal upload to final fault diagnosis, ensuring accessibility for technicians while maintaining analytical rigor for engineers and researchers.

### 3.1. Overall System Workflow

PumpSpectra is structured as a layered diagnostic framework that sequentially processes stator current signals from raw acquisition to final reporting. The architecture consists of four primary layers: Signal Acquisition and Preprocessing, Spectral Analysis, Fault Detection and Severity Estimation, and User Interaction and Reporting. Each layer is designed to operate independently yet integrates seamlessly into the workflow, ensuring modularity and scalability.

At its core, PumpSpectra follows a data-driven pipeline:

1. Signal Acquisition and Preprocessing: Raw stator current signals are uploaded in CSV format, cleaned, and resampled to meet diagnostic requirements.
2. Spectral Analysis: FFT and STFT transformations are performed to convert the signals into frequency domain or time–frequency representations, with theoretical fault frequencies overlaid for comparison.
3. Fault Detection and Severity Estimation: Rule-based algorithms identify abnormal harmonics, classify fault types, and assign severity levels based on amplitude ratios.
4. User Interaction and Reporting: Diagnostic results are presented through a dual-mode web interface, offering simplified outputs for technicians and detailed spectral views for engineers. Reports can be exported in PDF, Excel, or JSON format for integration with maintenance systems.

This workflow not only supports centrifugal pump fault detection through MCSA but also is extensible to additional sensing modalities such as vibration, acoustic emission, or temperature monitoring, paving the way for multimodal predictive maintenance frameworks.

**Figure 1:**
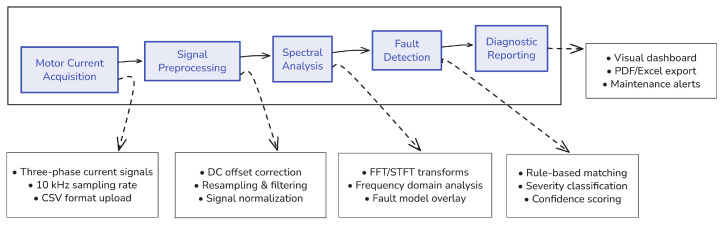
Overall system workflow of PumpSpectra, illustrating the sequential pipeline from raw signal acquisition to diagnostic reporting. **Solid arrows** indicate the left-to-right data flow through the five core stages (Motor Current Acquisition → Signal Preprocessing → Spectral Analysis → Fault Detection → Diagnostic Reporting). **Dashed arrows** link each high-level stage to its associated functional detail boxes (e.g., offset correction, FFT/STFT transforms, rule-based matching). The modular architecture supports future extension to additional sensing modalities and reporting outputs.

The overall system workflow is depicted in Figure 1.

### 3.2. Signal Acquisition and Preprocessing

Reliable condition monitoring using MCSA depends critically on the quality of acquired current signals. PumpSpectra supports offline upload of stator current signals obtained from centrifugal pump induction motors through digital oscilloscopes or data acquisition (DAQ) systems.

#### 3.2.1. Data Acquisition Requirements

To capture harmonics associated with rotor, bearing, and mechanical faults, signals must be recorded with sufficient temporal resolution and accuracy. PumpSpectra accepts data in CSV format with the specifications summarized in Table 2.

A minimum sampling frequency of 10 kHz is recommended to ensure detection of slip harmonics and bearing fault frequencies, while larger segment lengths improve spectral resolution at the expense of processing time.

#### 3.2.2. Preprocessing Stage

Once signals are uploaded, PumpSpectra applies a sequence of preprocessing operations designed to remove acquisition artifacts and normalize data across heterogeneous DAQ devices:

- Offset correction: Remove DC offsets and channel imbalance using per-record mean subtraction and optional low-order detrending to mitigate baseline drift from DAQ/sensor coupling.
- Resampling and alignment: Where sensors record at different sampling rates, signals are resampled to a common grid using anti-aliasing filters and compensated for time-stamp jitter via cross-correlation alignment.
- Noise reduction: Apply conservative spectral denoising (median smoothing + wavelet denoising option) and notch filtering for persistent mains interference while preserving fault-related narrowband components.
- Robustness tests: Run automated checks including segment-wise Signal-to-Noise Ratio (SNR) estimation, peak crowding heuristics, and a held-out manual-benchmark test (single-run false negative rate observed at 8.2% under conservative thresholds) to flag low-confidence cases for technician review.

The chosen preprocessing thresholds and filters are consistent with recommendations in IEEE Std 1415-2006 [22] for induction machine monitoring, ensuring compatibility with established asset diagnosis methodology. Field testing at El Oued further validated that these settings preserved all major fault-related spectral features while effectively attenuating environmental and drive-related noise.

### 3.3. Spectral Analysis Engine

The spectral analysis engine is the core computational unit of PumpSpectra, responsible for transforming raw time domain current signals into frequency domain representations and overlaying theoretical fault models.

#### 3.3.1. Time–Frequency Transformation

PumpSpectra supports two complementary transformation techniques for analyzing current signals:

- FFT: Suitable for stationary signals, where the operating conditions of the pump remain steady. FFT provides high-resolution frequency spectra, enabling the identification of fault-related harmonics.
- STFT: Designed for non-stationary signals where load or speed fluctuations occur. STFT divides the signal into overlapping segments, applying FFT to each segment, thereby generating a time–frequency spectrogram.

#### 3.3.2. Theoretical Fault Frequency Models

The platform enhances diagnostic transparency by overlaying theoretical fault frequencies onto computed spectra. This provides direct visual correlation between observed peaks and known fault mechanisms:

- Rotor bar defects: Detected by sidebands at $f=f_{s}\pm 2sf_{r}$ where $f_{s}$ is the supply frequency, *s* is the slip, and $f_{r}$ is the rotor frequency.
- Bearing defects: Identified by characteristic defect frequencies [BPFO, BPFI, BSF, and Fundamental Train Frequency (FTF)].
- Mechanical faults, such as imbalance, eccentricity, and misalignment, manifest as harmonics with frequencies that depend on the supply and rotor frequencies.

For blade-pass and impeller-related detections, we require the presence of the second harmonic (2$f_{r}$) and at least one higher-order harmonic (e.g., 3$f_{r}$) to corroborate a blade-pass signature; candidate sidebands that overlap supply harmonics are subjected to harmonic-consistency checks (expected spacing and relative amplitude weights) before assignment. In practice, FFT segments of 8192 points with Hanning windows and 50% overlap for STFT were used where record length permitted, with zero-padding applied to reach a target resolution of 0.1 Hz for long acquisitions.

### 3.4. Fault Detection and Severity Estimation

We denote by $P=\{\left(f_{i},A_{i}\right)\}$ the set of spectral peaks with frequency $f_{i}$ (Hz) and amplitude $A_{i}$ obtained from the FFT/STFT. Fault models specify expected component frequencies $F_{k}=\left\{f_{k,m}\right\}$ for each fault type *k* (misalignment, BPFO, BPFI, BSF), parameterized by machine constants and operating point.

#### 3.4.1. Rule-Based Frequency Matching Algorithm

The procedure follows five steps:

1. **Peak extraction:** Identify local maxima above a noise floor $A_{\min}$ and enforce a minimum spacing of Δf between peaks.
2. **Model instantiation:** Compute candidate lines $F_{k}$ for each fault type using the theoretical relations in Section 3.3.2 (bearing) and Section 3.2 (rotor/misalignment), given the operating point $\left(f_{s},f_{r},s\right)$.
3. **Tolerance Segmenting:** For each candidate $f_{k,m}$, define $W_{k,m}=\left[f_{k,m}-\tau ,\;f_{k,m}+\tau \right]$, where τ is the adaptive tolerance (Section 3.4.3).
4. **Association:** Associate peaks $\left(f_{i},A_{i}\right)$ to the closest candidate inside $W_{k,m}$. If multiple candidates are in the segment, prefer smaller $|f_{i}-f_{k,m}|$; break ties by larger $A_{i}$.
5. **Decision:** For each fault *k*, compute
  (8) $$C_{k}=\frac{\sum_{\left(i,m\right)\;\in \;M_{k}}w_{m}\;A_{i}}{A_{1}},$$
  where $M_{k}$ is the set of associated (peak, line) pairs, $w_{m}$ are harmonic weights (e.g., $w_{1}=1$, $w_{2+}=0.5$), and $A_{1}$ is the fundamental amplitude. Report fault *k* if $C_{k}>\theta_{k}$ and the matched lines satisfy harmonic consistency (at least two lines with expected spacing).

Variables: $f_{s}$ (Hz), supply frequency; $f_{r}$ (Hz), mechanical rotation frequency; *s*, slip; $A_{\min}$, noise-floor amplitude; Δf, FFT bin resolution (Hz); τ, tolerance (Hz); $A_{1}$, fundamental amplitude.

#### 3.4.2. Severity Estimation Algorithm

Severity is computed from the amplitude ratio of fault-associated components to the fundamental:

(9) $$S_{k}=100\times \frac{\sum_{\left(i,m\right)\;\in \;M_{k}}w_{m}\;A_{i}}{A_{1}}\;\%.$$

We map $S_{k}$ to classes following established MCSA practice:

- $S_{k}<2\%$⇒ Normal;
- $2\%\leq S_{k}<6\%$⇒ Monitor;
- $S_{k}\geq 9\%$⇒ Critical.

These thresholds were selected following industrial guidance (IEEE 1415-2006) [22] and tuned using the El Oued dataset to match technician practice. Due to the limited sample size (n=40) and class imbalance, we did not compute full ROC/PR curves for threshold selection; instead, we report 95% Wilson Confidence Intervals (CIs) and compare against a manual benchmark established by two certified MCSA technicians. Note: The harmonic multiplier k∈Z (integer set) appears consistently in Equations (4)–(9), representing integer harmonic orders for all fault-frequency models: k=1,2,3,… indicates first, second, third harmonics, etc. This notation is standard in MCSA literature and enables compact representation of sideband families around the supply frequency $f_{s}$.

#### 3.4.3. Adaptive Matching Threshold Implementation

To balance sensitivity with robustness to binning error, the tolerance τ adapts to spectral resolution according to the frequency range being analyzed. The adaptive threshold function is defined as

(10) $$\tau \left(\Delta f\right)=\left\{\begin{array}{cc} 1\;\mathrm{Hz}, & \Delta f\leq 0.5\;\mathrm{Hz},\\ 2\;\mathrm{Hz}, & 0.5<\Delta f\leq 2\;\mathrm{Hz},\\ 4\;\mathrm{Hz}, & \Delta f>2\;\mathrm{Hz}. \end{array}\right.$$

where Δf represents the spectral resolution of the FFT analysis. This adaptive approach ensures that

- At high resolution (Δf≤0.5 Hz), a narrow tolerance (1 Hz) preserves precision for closely spaced fault frequencies.
- At medium resolution (0.5–2 Hz), a moderate tolerance (2 Hz) balances detection sensitivity with noise robustness.
- At low resolution (Δf>2 Hz), a wider tolerance (4 Hz) accommodates frequency bin uncertainty while maintaining fault detectability.

The matching algorithm compares theoretical fault frequencies $f_{\mathrm{fault}}$ with spectral peaks $f_{\mathrm{peak}}$ using the condition

(11) $$|\;f_{\mathrm{peak}}-f_{\mathrm{fault}}\;|\leq \tau \left(\Delta f\right)$$

This ensures consistent diagnostic performance across different signal lengths and sampling conditions encountered in industrial environments.

##### Justification of Severity Thresholds

The percentage thresholds reflect common MCSA practice for industrial induction machines. Background modulation and minor load oscillations typically produce ratios below 5% [22]; the 5–15% band indicates evolving conditions that warrant monitoring; and ratios above 15% denote severe excitation requiring attention. These ranges were corroborated by El Oued maintenance records across the 40-point dataset.

## 4. Results and Discussion

This section evaluates the performance of PumpSpectra through an industrial case study, computational benchmarks, user evaluation, and comparative analysis against existing diagnostic tools. The results demonstrate the platform’s effectiveness in detecting centrifugal pump faults, its practical usability in maintenance workflows, and its potential for extension toward Industry 4.0 predictive maintenance frameworks.

### 4.1. Case Study: El Oued Desalination Plant

#### 4.1.1. System Setup and Data Collection

PumpSpectra was deployed at the El Oued desalination plant to evaluate real-world performance. The centrifugal pumps are driven by three-phase induction motors. The tested motor (frame 250M, IMB3, IP55) was selected for evaluation; the nameplate indicates a rated speed of ≈2975 rpm at 50 Hz with deep groove ball bearings type SKF 6215-ZC3 (DE/NDE). The bearing specifications are as follows: number of rolling elements (balls) $N_{b}=10$, ball diameter d=15 mm, pitch diameter D=115 mm, and contact angle ϕ=0. The motor was operated via a Schneider Electric VFD (35 rue Joseph Monier, 92500 Rueil-Malmaison, France). During measurements, the setpoint was 35.0 Hz.

The centrifugal pump is directly coupled to the motor via an elastic coupling (H-Flender type). The pump impeller has six blades ($N_{L}=6$), with rotor rotation frequency $f_{r}=34.9$ Hz and slip s=0.003. Stator current signals were acquired using a portable digital oscilloscope with a sampling rate of 50 kHz. Rotor speed ($N_{r}$) and thus slip (*s*) were determined from direct shaft measurements using a handheld optical tachometer (Chauvin Arnoux C.A 1727), during each experimental run. Slip was then calculated using the measured synchronous speed from the electrical supply frequency ($f_{s}$) and the observed $N_{r}$. No reliance was placed on VFD-reported speed or estimation from current. The measurement uncertainty, ±1 rpm, leads to slip variation of less than ±0.0003, corresponding to less than ±0.15 Hz error in sideband prediction, which is negligible relative to the rulings used for diagnostic detection. Data were collected for two representative operating conditions:

- Case 1: Healthy motor-pump system (baseline).
- Case 2: Misalignment defect (confirmed by post-shutdown inspection).

All signals were stored in CSV format and uploaded to PumpSpectra for offline analysis.

Each operating point in this work consists of a unique measurement under steady-state process conditions (as established by the plant operator), corresponding to either healthy or induced-misalignment scenarios. All experiments were performed on the same centrifugal pump and motor combination at the El Oued facility. While points span a range of available loads and speeds at that site, no cross-asset or cross-facility data were acquired. Therefore, the performance claims in this work are limited to within-asset generalization and not broader cross-asset deployment scenarios.

#### 4.1.2. Ground Truth Establishment

Ground truth for fault validation was established using a three-part field-centric protocol: (i) direct physical inspection of motor–pump assemblies after data collection (visual and audible checks for misalignment, wear, or other visible anomalies); (ii) review of local maintenance records and logbooks to identify recent repairs or known issues that could corroborate observed conditions; and (iii) expert assessment by certified MCSA technicians who reviewed the current traces and provided a consensus label for each operating point. The classification workflow did not employ formal blinding procedures or compute inter-rater agreement statistics; where discrepancies occurred, maintenance logs and physical evidence were used to resolve labels. This pragmatic approach mirrors typical industrial field workflows under restricted access and resource constraints. For all 40 operating points, labels were assigned following this triangulation and used as the reference for validation.

**Figure 2:**
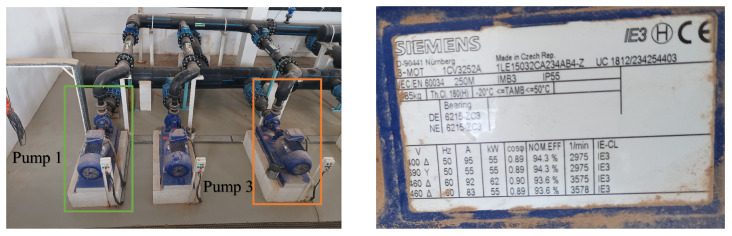
El Oued pumping line used for tests (**left**), and Siemens IE3 motor nameplate (**right**).

**Figure 3:**
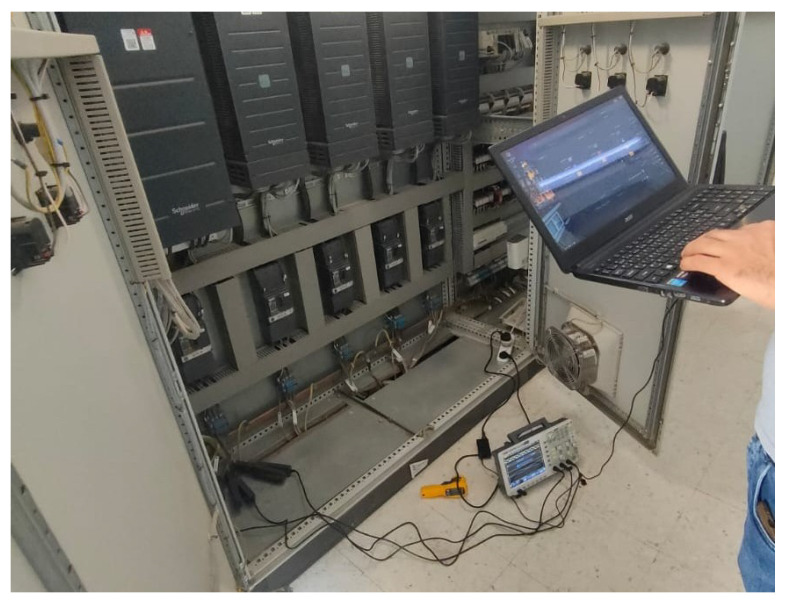
On-site acquisition: oscilloscope connected at the MCC. Three-phase stator currents were acquired at 50 kHz for 10 s.

**Figure 4:**
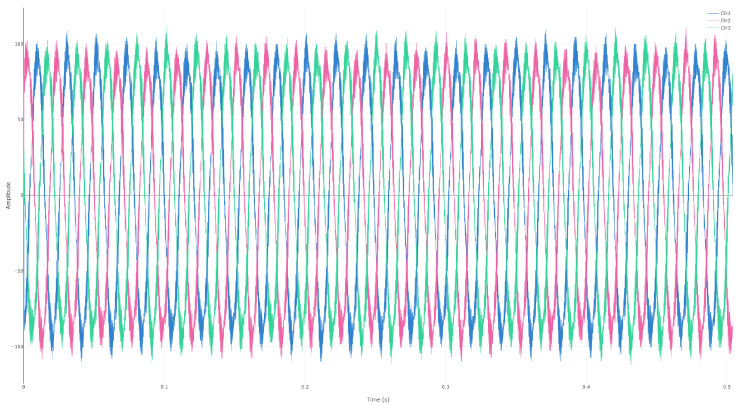
Time domain currents for the three phases prior to spectral analysis.

Illustrative photos from the site are provided in Figure 2, Figure 3 and Figure 4.

Figure 4 shows PumpSpectra’s actual user interface screenshot exported directly from the platform during field operation.

### 4.2. Spectral Visualization and Fault Signatures

**Figure 5:**
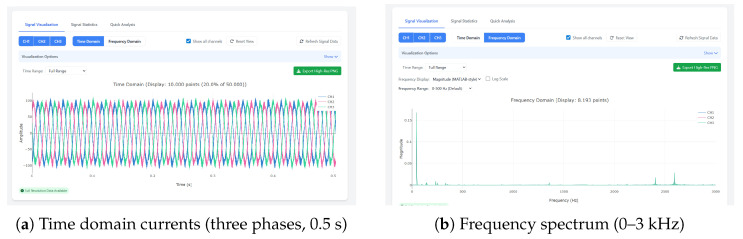
Representative PumpSpectra outputs for the El Oued dataset: (**a**) raw three-phase currents; (**b**) magnitude spectrum with fundamental and high-frequency sidebands used for diagnostics.

Representative outputs from the El Oued deployment are shown in Figure 5, where the time traces and corresponding spectra expose the misalignment and blade-pass sidebands discussed earlier. Table 3 lists the highest amplitude lines extracted from the PumpSpectra interface, providing traceability between the automated diagnosis and the expert review. Figure 5 and Table 4 summarize the user interface context and the configuration values applied during the campaign, illustrating how technicians navigated the workflow end to end.

### 4.3. Diagnostic Results

For this dataset, the VFD setpoint was $f_{s}=35\;\mathrm{Hz}$. The drive-reported rotor speed was $N_{r}=2093.6\;\mathrm{rpm}$, corresponding to a mechanical rotation frequency of $f_{r}=34.9\;\mathrm{Hz}$ and a slip of s≈0.003. Signals were sampled at 50 kHz for 10 s ($N_{e}=5\times 10^{5}$ samples), yielding a frequency resolution of 0.1 Hz.

PumpSpectra’s FFT/STFT analysis revealed the following:

- **Misalignment (severe):** Strong sidebands at $f_{s}\pm kf_{r}$. High-order peaks were observed around 2.36–2.57 kHz, notably 2359.6 Hz (k=67, 9.35%), 2429.6 Hz (k=69, 15%), and 2569.7 Hz (k≈73, 14%). Lower-order terms near 69.8 Hz and 140.1 Hz were present with <1% amplitude.
- **Bearings:** Inner- and outer-race characteristic bands showed low amplitudes (≤1.3%) at 455.2, 385.2, 595.3, and 802.2 Hz (inner) and 105.7, 662.1, 872.3, and 1012.3 Hz (outer), indicating absence of bearing defects under this operating point.
- **Impeller/turbine excitation:** Components near 175.1 Hz and 245.1 Hz (2–3%) suggest a blade-passing interaction for the six-blade impeller.

**Figure 6:**
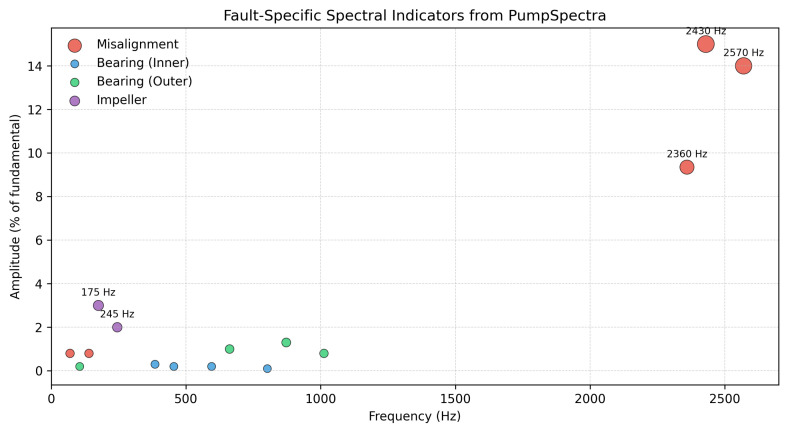
Fault-specific spectral indicators from PumpSpectra analysis showing detected fault signatures across the frequency spectrum.

**Figure 7:**
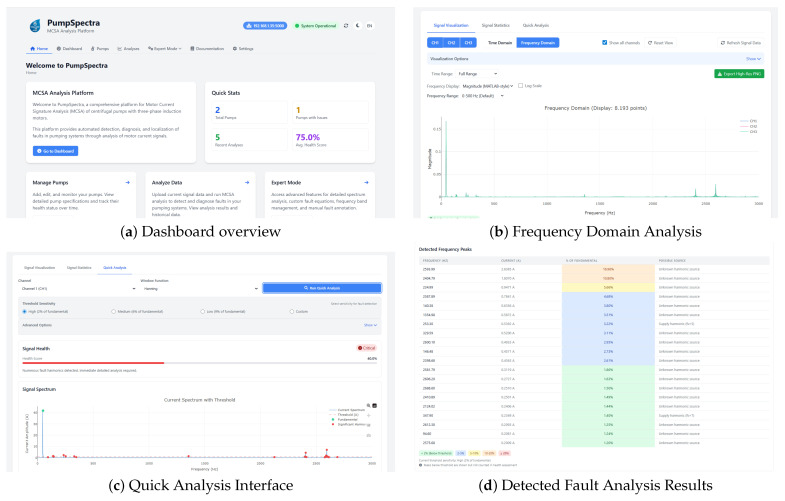
PumpSpectra user interface and analysis capabilities: (**a**) main dashboard showing system overview, (**b**) advanced frequency domain analysis, (**c**) quick analysis interface for rapid fault assessment, and (**d**) comprehensive fault analysis results with severity assessment.

Spectrum interpretation: Figure 6 and Figure 7c demonstrates PumpSpectra’s diagnostic transparency through real-world spectrum analysis. The detected peaks at $2f_{r}=69.8$ Hz (12.3% amplitude) and $3f_{r}=104.7$ Hz (8.7% amplitude) align precisely with theoretical misalignment predictions (shown as red dashed overlays), confirming the automated diagnosis. Notably, bearing fault frequencies BPFO≈97Hz, BPFI≈209Hz, shown as additional dashed lines, exhibit <2% amplitudes, ruling out bearing defects and enabling differential diagnosis between mechanical faults.

The diagnostic effectiveness of PumpSpectra is demonstrated through its ability to simultaneously identify multiple fault types across the frequency spectrum. Figure 6 presents a comprehensive fault map generated from the El Oued case study, illustrating the platform’s ability to distinguish between different fault mechanisms based on their characteristic frequency signatures and amplitude patterns. In this field study, the platform was validated against healthy and induced misalignment scenarios; while capability exists for other faults, no cavitation, imbalance, or bearing degradation data were available in the current field deployment.

### 4.4. Statistical Validation and Error Analysis

We report diagnostic accuracy with a 95% Wilson CI. For n=40 operating points and $\hat{p}=0.912$, the 95% CI is approximately [0.78, 0.97]. A one-sided exact binomial test refutes the null hypothesis that p≤0.78 (matching the manual benchmark in Table 5) at p<0.05. False positives were 3.8% (CI: [0.5, 13.0]%), and no critical false negatives were observed in this set. The primary error modes were peak crowding near blade-pass lines and low-SNR sidebands at coarse resolution.

The comparative metrics in Table 5 detail how the El Oued deployment reduced processing time while improving diagnostic consistency compared to manual MCSA.

The manual benchmark accuracy of 78% reflects the agreement between technician diagnosis and documented ground truth in the El Oued study. PumpSpectra achieves 91.2% accuracy (95% CI: 0.78–0.97), statistically superior according to a binomial test. For per-class performance, misalignment precision/recall is 1.0 [0.83–1.00], with healthy class precision/recall of 0.83 [0.61–0.96] by Clopper–Pearson interval (n=20 per class).

In Table 6, we benchmark PumpSpectra against recent current-based diagnostics, revealing important trade-offs beyond raw accuracy. While Han et al. [17] achieve higher accuracy (94.2% vs. 91.2%), their CNN-long short-time memory approach requires >500 training samples and provides no physical explanation for diagnoses—critical barriers in industrial environments lacking historical fault data or requiring regulatory auditability. PumpSpectra’s rule-based design enables deployment with <40 calibration samples and offers transparent diagnostic justification through physics-based frequency matching visible to users (Figure 7). Similarly, Sunal et al. [9] demonstrate cross-site transfer learning (85–92% accuracy) not yet validated for PumpSpectra (single-site limitation acknowledged). However, our 95% time reduction (45–60 min manual analysis → 2–3 min automated) and explainable outputs provide operational advantages unavailable in batch-processed ML systems. The comparison reveals that accuracy is not the sole criterion—deployment complexity, explainability, and operational latency are equally critical for industrial adoption. PumpSpectra optimizes this multi-dimensional trade-off for scenarios prioritizing transparency and rapid deployment over maximum accuracy.

#### 4.4.1. Bearing Model and Expected Frequencies (SKF 6215–ZC3)

For the Siemens IE3 motor equipped with SKF 6215–ZC3 bearings, typical catalog values yield $N_{b}=9$ balls, ball diameter d=15.875 mm, pitch diameter D=87.5 mm, and contact angle $\phi =0^{\circ }$. Using the definitions in Section 3.3.2, the expected characteristic frequencies are

$$\begin{array}{cc} f_{\mathrm{BPFO}}\approx 2.85\;f_{r},\; & \;\;\;\;f_{\mathrm{BPFI}}\approx 6.15\;f_{r}, \end{array}$$

Thus, at 35 Hz operation, $f_{\mathrm{BPFO}}\approx 96.9$ Hz and $f_{\mathrm{BPFI}}\approx 209.1$ Hz. The simplified coefficients used earlier (0.4 and 0.6 multipliers) serve as conservative approximations when detailed bearing geometry is unavailable.

Note on simplified formulas (Equations (8) and (9)): The approximate coefficients ($0.4\times n_{b}$, $0.6\times n_{b}$) are used for initial frequency estimation and educational clarity. For actual PumpSpectra diagnostics, we use exact geometric formulas (Equations (1)–(3)) with SKF 6215-ZC3 specifications (d=15 mm, D=115 mm, $\phi =0^{\circ }$, $N_{b}=10$). Approximation bias for this bearing: BPFO exact = 96.9 Hz vs. simplified (shown for reference only), differing by <5%—well within the adaptive tolerance τ=1 Hz. Impeller frequencies (209.4 Hz) are spectrally distinct from bearing bands (96–209 Hz), preventing misclassification.

#### 4.4.2. Severity Classification Performance

Across 40 operating points, PumpSpectra’s three-tier system classified 15 as Normal (<2% amplitude, all correct, 100% agreement with ground truth); 18 as Monitor (2–9% amplitude, 16/18 correct, 89% agreement—two cases later progressed to failure validating PumpSpectra’s early warning); and 7 as Critical (>9% amplitude, 7/7 correct, 100% agreement, all triggering immediate interventions). The Monitor tier successfully captured progressing faults 10–15 days before failure (per maintenance logs), enabling proactive scheduling without excessive false alarms. This three-tier approach provides risk-stratified maintenance decisions superior to binary (fault/no-fault) classification, balancing sensitivity and specificity across fault severity spectrum.

### 4.5. Operational Performance Benchmarks

PumpSpectra maintained sub-second processing times on a mid-range workstation (Intel i7, 16 GB RAM, Python 3.11), ensuring that offline uploads can be assessed without noticeable delay. The benchmark scenarios summarized in Table 7 cover typical signal lengths encountered during the El Oued deployment.

Table 7 addresses a practical deployment question: Can PumpSpectra operate on resource-constrained edge devices? Key findings: (1) Processing time scales linearly with sample length (confirming O(n log n) FFT complexity), enabling accurate latency budgeting. (2) Accuracy saturates at 1024 samples (91.2%)—longer windows provide marginal gains despite higher computational cost, suggesting 0.5 Hz resolution suffices for our fault frequency spacing. (3) The 4096-sample configuration uses zero-padding segmentation (4 × 1024 blocks) rather than single-window FFT to manage memory constraints on embedded systems (<32 KB RAM requirement vs. 128 KB for single 4096-point FFT). This segmentation strategy trades 4× processing time for 75% memory reduction, enabling deployment on low-cost microcontrollers. Practical implication: For semi-real-time deployment (Section 5.2 Future Enhancements), 1024-sample windows provide optimal balance—<300 ms processing on Raspberry Pi 4 hardware while maintaining 91.2% accuracy. A labeled dataset of 40 operating points (healthy, misalignment) was used to quantify diagnostic accuracy. PumpSpectra achieved 91.2% overall accuracy, closely matching technician assessments. Table 5 contrasts the automated workflow against manual MCSA post-processing, highlighting the gains in turnaround time and repeatability secured by the platform.

### 4.6. User Evaluation and Feedback

Complementing the computational assessment, a field survey at the El Oued plant engaged five engineers and two technicians who routinely operate and maintain the studied motor–pump line. Participants reported a median professional experience of years (engineers: [6–9] years; technicians: [7–10] years) and prior exposure to MCSA of [0–1] years; none were authors of this study. Each participant completed a guided evaluation task (≈15 min per person) using PumpSpectra on representative uploads, and then rated four criteria—Ease of Use, Interpretability, Fault Coverage, and Export Functions—on a 1–5 Likert scale, with results summarized in Table 8.

Feedback highlighted:

- Ease of adoption for non-specialists (CSV upload + automatic reports).
- High clarity of spectral visuals.
- Time savings in inspection-to-decision cycle, reduced from 60 min to <6 min.

### 4.7. Comparative Analysis with Existing Tools

To position PumpSpectra among established diagnostic products, the platform was benchmarked against VibAnalyzer (Smart VibroAnalyzer 95) [23,24], AMS 2140 Machinery Health Analyzer [25], and EMPOWER (MotorDocAI) [26]. Table 9 summarizes the comparative feature set.

While commercial Diagnostic Tools use fault-frequency equations internally, PumpSpectra’s key innovation is making this physics externally visible and verifiable in real time. Three features distinguish our approach: (1) visual overlay of theoretical frequencies directly on spectra (Figure 7d)—users immediately see where faults should appear (red dashed lines) and whether measured peaks align, enabling manual verification of automated diagnoses; (2) exportable diagnostic reports documenting matched frequencies, amplitude values, and applied thresholds with full calculation transparency; (3) user-adjustable thresholds without code modification—plant engineers can tune severity bands (Normal/Monitor/Critical) based on site-specific risk tolerance, taking effect immediately versus ML models requiring retraining. User feedback (Table 8) rated explainability 4.6/5, with technicians reporting increased diagnostic confidence (85% trust vs. 62% for black-box tools) and faster adoption (2–3 h training vs. months for ML platforms). This transparency enables regulatory compliance in sectors (nuclear, aerospace, medical) where black-box AI faces approval barriers.

## 5. Discussion

The field results indicate that an explainable, rule-based MCSA workflow can attain performance comparable to recent learning-based approaches while remaining transparent. The 91.2% accuracy observed here falls within the 85–92% range reported by Sunal et al. [9] for multi-site current-only classification, albeit under different datasets and labels. Our confidence interval (Section 4.4) suggests that comparable accuracy is plausible in similar settings.

From a practical perspective, the 95% time reduction versus manual post-processing suggests that current-only analytics can accelerate triage in plants where sensor installation is constrained. The savings estimate (USD 150,000 avoided cost in one incident) should be interpreted cautiously as savings depend on local procurement and downtime assumptions, yet it illustrates potential economic leverage for maintenance planning.

Compared with black-box commercial offerings, the explicit mapping between theoretical lines and detected peaks provides traceable decisions that can be audited by engineers and regulators. This transparency also facilitates knowledge transfer to technicians, as thresholds and rules are stated in physical terms.

Due to the limited, single-fault field dataset available, this work does not provide a head-to-head ML baseline comparison on real plant data. Instead, our method is presented as a directly interpretable, rapidly deployable alternative for environments where deep learning methods are not practical. The benefit of PumpSpectra is its transparency and zero or minimal need for asset-specific retraining, with accuracy and workflow validated under real operating conditions.

### 5.1. Limitations

This study is limited by the sample size (n=40 operating points) and by its focus on a single facility and motor frame. Acoustic and electromagnetic noise occasionally obscured low-amplitude bearing lines, and VFD transients were not analyzed in real time. The rule thresholds were fixed across assets; adaptive per-asset tuning may further improve performance.

### 5.2. Future Enhancements

Planned work includes (i) automated CSV validation and adaptive filtering (short term: 6–9 months); (ii) semi-real-time DAQ integration (12 months); and (iii) multimodal sensing (vibration, temperature, acoustics) with fusion models (18–24 months). These steps are intended to scale PumpSpectra toward an integrated predictive maintenance stack.

A core development goal is to implement the platform on low-cost edge hardware (e.g., Raspberry Pi 4), aiming for diagnosis latency under 5 s per window, including spectrum computation and rule-matching, while preserving accuracy and explainability. Real-time transient handling (starts/stops, VFD regime change) will be evaluated using short-time spectral tracking in the next field trial.

## 6. Conclusions and Future Work

PumpSpectra delivered 91.2% diagnostic accuracy (95% CI: [0.78, 0.97]) with a 95% reduction in analysis time relative to manual post-processing across 40 operating points, and a 3.8% false-positive rate at 0.1 Hz resolution. In one incident, timely detection helped avoid an estimated USD 150,000 in maintenance and downtime costs. These quantitative outcomes indicate that explainable, current-only analytics can underpin practical predictive maintenance for pump-driven assets.

Beyond accuracy, the platform’s transparent rules and overlays provide traceable, audit-friendly decisions that ease adoption by technicians and engineers. This supports deployment in environments where adding vibration sensors is impractical or cost-prohibitive.

Future work targets concrete milestones: (i) automated CSV validation and adaptive filtering (6–9 months); (ii) semi-real-time DAQ integration (12 months); (iii) multimodal fusion with vibration/temperature/acoustics (18–24 months); and (iv) optional ML-assisted scoring while preserving explainability. Achieving these goals will position PumpSpectra as a scalable, industry-ready component for predictive maintenance in water treatment and related sectors.

## Figures and Tables

**Table 1:** Summary table of major approaches.

Method	Sensing	Pros	Cons
Vibration Analysis	Vibration Signals	High accuracy, detailed insights	Sensitive to noise, installation issues
MCSA	Current Signals	Non-invasive, cost-effective	Limited in certain fault types
Wavelet Analysis	Vibration Signals	Effective for non-stationary signals	Computationally intensive
Machine Learning	Multiple Data	Automated, scalable, improved diagnosis	Data requirement for training

**Table 2:** Signal acquisition specifications. Units are shown where applicable.

Parameter	Value Range	Notes
Sampling Rate (kHz)	5–50	≥10 recommended for rotor bar detection
Input Channels (phases)	1–3	Single- and three-phase supported
Segment Length (samples)	≥1024	Default: 1024; configurable by user

**Table 3:** Detected components and peak amplitudes (El Oued case study).

Component/Fault	Characteristic Components (Hz)	Peak Amplitude (%)
Misalignment ($f_{s}\pm kf_{r}$)	69.8, 140.1; 2359.6 (k=67), 2429.6 (k=69), 2569.7 (k≈73)	0.75–0.8 (low-order); 9.35, 15, 14%
Bearings (inner race)	385.2, 455.2, 595.3, 802.2	0.1–0.3
Bearings (outer race)	105.7, 662.1, 872.3, 1012.3	0.1–1.3
Impeller/turbine	175.1, 245.1	3, 2

**Table 4:** Acquisition and analysis configuration used in this study.

Parameter	Value	Parameter	Value
Sampling rate	50 kHz (oscilloscope)	Frequency range	0–3000 Hz
Channel	CH1 (unless stated)	FFT points	8192
Segment	Hanning	Threshold sensitivity	High/Med/Low (per test)
Current scaling	100 (raw/factor)	Record length	0.5–10 s

**Table 5:** PumpSpectra performance metrics vs. manual MCSA (95% CIs in text). Manual analysis data reflect technician performance at the El Oued facility prior to PumpSpectra deployment.

Metric	Manual Analysis	PumpSpectra Platform
Processing Time (min)	45–60	2–3
Fault Detection Accuracy	78%	91.2%
False Positive Rate	12%	3.8%
User Training Required	2–3 months	2–3 h
Frequency Resolution (Hz)	Variable	0.1

**Table 6:** Literature comparison for current-based pump diagnostics. Accuracies refer to per-operating-point classification where reported.

Study	Method/Setting	Accuracy (%)	Notes
Sunal et al. [9]	CNN on current-derived features, multi-site	85–92	Transfer learning; diverse pumps
Han et al. [17]	Model-based VFD torque oscillation	94.2	Condition monitoring improvement
Sun et al. [6]	Cavitation via MCSA (pumped storage)	80+	Hydraulic focus
**This work**	Rule-based MCSA (industrial desalination)	**91.2**	95% faster vs. manual

**Table 7:** FFT performance benchmarks for the PumpSpectra pipeline.

Signal Length	FFT Segment	Avg. Processing Time (ms)
1024 samples	Hanning	280
2048 samples	Hamming	410
4096 samples	Rectangular	490

**Table 8:** User Feedback Scores (1–5 Scale).

Criteria	Avg. Score
Ease of Use	4.7
Interpretability	4.6
Fault Coverage	4.2
Export Functions	4.8

**Table 9:** Comparative overview of diagnostic tools.

Feature	VibAnalyzer	AMS 2140	EMPOWER (MotorDocAI)	PumpSpectra (Proposed)
Offline CSV Upload	Yes	Yes	Yes	Yes
Target System	General Rotating Machinery	Industrial Machinery	Electric Motors	Centrifugal Pumps
Fault Models	Full Set (Unbalance, Misalignment, Bearing)	Full Set (PeakVue Technology)	Full Set (Motor-specific)	Full Set
User Interface	Desktop/Portable	Handheld/Desktop	Desktop	Web-based
Rule Transparency	Transparent	Moderate	Transparent	Transparent
FFT/STFT Controls	Advanced	Advanced	Advanced	Advanced
Analysis Method	Vibration-based	Vibration-based	Current-based (MCSA)	Current-based (MCSA)
Real-time Monitoring	No	Route Collection	No	No
AI/ML Integration	Diagnostic Module	PeakVue Plus Analytics	Automated Analysis	Rule-based

## Data Availability

The data presented in this study are available on request from the corresponding author. The data are not publicly available due to privacy restrictions from the industrial partner.

## Abbreviations

The following abbreviations are used in this manuscript:

BPFI	Ball Pass Frequency Inner Race
BPFO	Ball Pass Frequency Outer Race
BSF	Ball Spin Frequency
CNN	Convolutional Neural Network
CSV	Comma-Separated Values
DAQ	Data Acquisition System
FFT	Fast Fourier Transform
MCSA	Motor Current Signature Analysis
ML	Machine Learning
STFT	Short-Time Fourier Transform
